# Supplementary material for: Pharmaceutical expenditure changes under the volume-based procurement policy: Effects and influencing factors
Source: PLoS One. 2025 Aug 14;20(8):e0330296. doi: 10.1371/journal.pone.0330296 (PMC12352851; doi:10.1371/journal.pone.0330296)
Supplement: S1 Table — GDP, gross domestic product; CNY, Chinese yuan. (PDF) [file pone.0330296.s001.pdf]

S1 Table. General information of observation regions.

Regions		Per capita GDP (CNY)	Population size (million)	Number of medical institutions (per 1000 population)	Number of hospital beds (per 1000 population)	Number of skilled health workers (per 1000 population)	Number of licensed (assistant) doctors (per 1000 population)	Per capital health expenditure (CNY)	Annual average clinical visits	Annual hospitalization rate (%)
Pilot cities	Xiamen	142739.00	4.29	0.49	4.38	8.61	3.54	4276.70	6.27	15.30
	Shanghai	157279.00	24.28	0.23	6.03	8.40	3.10	10430.53	11.35	18.70
	Tianjin	90371.00	15.62	0.38	4.37	7.00	3.00	6233.15	7.87	10.90
	Dalian	99996.00	5.99	0.69	8.22	9.58	3.85	4160.65	4.59	16.30
	Shenyang	77777.00	7.55	0.69	9.52	10.82	4.21	4160.65	4.59	16.30
	Chongqing	75828.00	31.24	0.67	7.42	7.20	2.70	4530.36	5.62	24.10
	Xi'an	92256.00	10.20	0.69	7.11	11.00	10.65	4706.80	5.39	21.10
Expansion provinces	Jiangsu	123607.00	80.70	0.43	6.39	7.80	3.20	5525.61	7.65	18.90
	Inner Mongolia	67852.00	25.40	0.97	6.34	7.70	3.10	4604.72	4.21	14.30
	Hubei	77387.00	59.27	0.60	6.80	7.00	2.60	4354.57	5.97	23.10
	Hunan	57540.00	69.18	0.83	7.32	7.30	2.80	4006.25	4.06	23.40
	Jilin	43475.00	26.91	0.82	6.33	7.00	2.90	4356.76	4.10	15.00
	Heilongjiang	36183.00	37.51	0.54	7.00	6.30	2.50	4027.08	3.00	16.10
	Guizhou	46433.00	36.23	0.79	7.31	7.40	2.50	3838.96	4.85	23.70
	Qinghai	48981.00	6.08	1.07	6.82	7.80	2.90	5843.23	4.38	17.40

Note: GDP, gross domestic product; CNY, Chinese yuan.
