# Supplementary material for: Pharmaceutical expenditure changes under the volume-based procurement policy: Effects and influencing factors
Source: PLoS One. 2025 Aug 14;20(8):e0330296. doi: 10.1371/journal.pone.0330296 (PMC12352851; doi:10.1371/journal.pone.0330296)
Supplement: S2 Table — (PDF) [file pone.0330296.s002.pdf]

S2 Table. General information of included drugs.

Anatomical category	Therapeutic category	Name of VBP drugs	Name of alternative drugs
C_Cardiovascular system	Antihypertensive drugs	Amlodipine<br>Losartan<br>Irbesartan<br>Irbesartan and Hydrochlorothiazide<br>Fosinopril<br>Lisinopril<br>Enalapril	Alisartan, Amlodipine Atorvastatin, Amlodipine Benazepril, Amlodipine Folic Acid, Olmesartan Amlodipine, Olmesartan Hydrochlorothiazide, Benazepril Hydrochlorothiazide, Levamlodipine, Felodipine, Compound Captopril, Captopril, Candesartan Cilexetil and Hydrochlorothiazide, Lisinopril Hydrochlorothiazide, Ramipril, Losartan Hydrochlorothiazide, Enalapril Folic Acid, Perindopril Amlodipine, Perindopril, Perindopril Indapamide, Telmisartan, Telmisartan Hydrochlorothiazide, Nifedipine, Valsartan Amlodipine, Valsartan, Valsartan Hydrochlorothiazide, Benazepril
	Lipid modifying agents	Atorvastatin<br>Rosuvastatin	Fluvastatin, Lovastatin, Pitavastatin, Pravastatin, Xuezhikang, Ezetimibe and Simvastatin, Zhibitai, Zhibituo
N_Nervous system	Antiepileptics	Levetiracetam	Oxcarbazepine, Magnesium Valproate, Sodium Valproate, Carbamazepine, Lamotrigine, Topiramate
	Psycholeptics	Olanzapine<br>Risperidone<br>Dexmedetomidine	Aripiprazole, Amisulpride, Loxapine, Perphenazine, Haloperidol, Quetiapine, Ziprasidone, Clozapine, Midazolam, Paliperidone, Sulpiride, Penfluridol, Tiapride, Chlorpromazine
	Psychoanaleptics	Escitalopram<br>Paroxetine	Fluvoxamine, Fluoxetine, Bupropion, Duloxetine, Fluoxetine, Trazodone, Sertraline, Venlafaxine
L_Antineoplastic and immunomodulating agents	Antineoplastic agents	Gifitinib<br>Imatinib<br>Pemetrexed Disodium	Dasatinib, Osimertinib, Afatinib, Nilotinib, Icotinib, Erlotinib
J_Antiinfectives for systemic USE	Antibacterials for systemic use	Cefuroxime	Cefprozil, Cefadinib, Cefaclor, Cefixime, Cefadroxil
	Antivirals for systemic use	Entecavir<br>Tenofovir Disoproxil	Propofol Tenofovir, Lamivudine, Telbivudine

A_Alimentary tract and metabolism	Antidiarrheals	Montmorillonite	Tannate Albumin, Loperamide, Berberine
B_Blood and blood forming organs	Antithrombotic agents	Clopidogrel	Aspirin, Ticagrelor, Cilostazol, Ticlopidine
M_Musculo-skeletal system	Antiinflammatory and antirheumatic products	Flurbiprofen	Piroxicam, Potassium Diclofenac, Sodium Diclofenac, Precoxib
R_Respiratory system	Drugs for obstructive airway diseases	Montelukast	Pemirolast, Pranlukast, Seratrodast
