# Supplementary material for: Pharmaceutical expenditure changes under the volume-based procurement policy: Effects and influencing factors
Source: PLoS One. 2025 Aug 14;20(8):e0330296. doi: 10.1371/journal.pone.0330296 (PMC12352851; doi:10.1371/journal.pone.0330296)
Supplement: S3 Table — VBP, volume-based procurement; INN, international nonproprietary name. GR1, the increment in 2019 against 2018; GR2, the increment in 2020 against 2019. (PDF) [file pone.0330296.s003.pdf]

S3 Table. Drug expenditure changes by each pilot city.

Pilot cities	VBP INNs					Alternative INNs					All observed drugs				
	Mar-Dec 2018	Mar-Dec 2019	Mar-Dec 2020	GR_1	GR_2	Mar-Dec 2018	Mar-Dec 2019	Mar-Dec 2020	GR_1	GR_2	Mar-Dec 2018	Mar-Dec 2019	Mar-Dec 2020	GR_1	GR_2
Shanghai	3.50	1.51	1.20	-56.93	-20.57	2.94	3.38	3.34	15.00	-1.00	6.43	4.88	4.54	-24.11	-7.04
Xiamen	0.21	0.12	0.11	-40.17	-10.32	0.15	0.21	0.24	39.22	17.02	0.36	0.33	0.35	-6.97	6.79
Dalian	0.21	0.15	0.10	-29.81	-32.58	0.14	0.18	0.17	29.36	-5.67	0.35	0.33	0.27	-6.36	-17.85
Tianjin	1.25	0.73	0.54	-41.41	-26.65	0.80	1.12	1.23	40.84	9.83	2.05	1.86	1.77	-9.34	-4.55
Shenyang	0.36	0.20	0.15	-43.32	-27.97	0.24	0.33	0.33	34.13	2.44	0.60	0.53	0.48	-11.85	-9.17
Xi’an	0.30	0.22	0.16	-26.84	-27.36	0.20	0.27	0.26	34.08	-2.46	0.50	0.49	0.42	-2.70	-13.76
Chongqing	0.80	0.54	0.42	-31.77	-23.12	0.55	0.77	0.83	41.85	7.27	1.34	1.32	1.25	-1.87	-5.28
Total	6.63	3.48	2.67	-47.46	-23.26	5.01	6.26	6.42	24.85	2.55	11.64	9.74	9.09	-16.32	-6.68

Note: VBP, volume-based procurement; INN, international nonproprietary name. GR_1, the increment in 2019 against 2018; GR_2, the increment in 2020 against 2019
