# Supplementary material for: Pharmaceutical expenditure changes under the volume-based procurement policy: Effects and influencing factors
Source: PLoS One. 2025 Aug 14;20(8):e0330296. doi: 10.1371/journal.pone.0330296 (PMC12352851; doi:10.1371/journal.pone.0330296)
Supplement: S4 Table — VBP, volume-based procurement; INN, international nonproprietary name. GR1, the increment in 2019 against 2018; GR2, the increment in 2020 against 2019. (PDF) [file pone.0330296.s004.pdf]

S4 Table. Drug expenditure changes by each expansion province.

Expansion provinces	VBP INNs					Alternative INNs					All observed drugs				
	Jan-Nov 2018	Jan-Nov 2019	Jan-Nov 2020	GR_1	GR_2	Jan-Nov 2018	Jan-Nov 2019	Jan-Nov 2020	GR_1	GR_2	Jan-Nov 2018	Jan-Nov 2019	Jan-Nov 2020	GR_1	GR_2
Inner Mongolia	0.59	0.62	0.27	4.78	-56.43	0.31	0.37	0.44	21.88	18.98	0.90	1.00	0.72	10.60	-28.15
Jilin	0.58	0.71	0.30	22.04	-57.25	0.29	0.34	0.38	17.20	12.54	0.87	1.05	0.69	20.43	-34.64
Jiangsu	3.49	4.14	1.41	18.62	-65.87	2.97	3.78	2.69	27.41	-28.85	6.46	7.93	4.10	22.65	-48.21
Hubei	1.19	1.19	0.55	0.09	-54.06	0.83	0.85	0.77	3.47	-9.35	2.02	2.05	1.32	1.47	-35.41
Hunan	1.14	1.43	0.60	25.58	-58.31	0.80	1.07	0.98	34.15	-9.20	1.94	2.50	1.57	29.12	-37.25
Guizhou	0.37	0.69	0.31	86.70	-54.71	0.25	0.41	0.46	63.62	14.41	0.62	1.10	0.78	77.42	-29.07
Qinghai	0.07	0.10	0.03	40.03	-70.78	0.05	0.07	0.10	58.32	33.33	0.12	0.17	0.13	47.11	-27.42
Heilongjiang	0.43	0.77	0.35	81.68	-54.74	0.12	0.41	0.40	237.39	-3.50	0.55	1.19	0.75	116.28	-36.98
Total	7.87	9.67	3.83	22.84	-60.41	5.61	7.31	6.23	30.43	-14.88	13.48	16.98	10.05	26.00	-40.80

Note: VBP, volume-based procurement; INN, international nonproprietary name. GR_1, the increment in 2019 against 2018; GR_2, the increment in 2020 against 2019
