# Supplementary material for: Pharmaceutical expenditure changes under the volume-based procurement policy: Effects and influencing factors
Source: PLoS One. 2025 Aug 14;20(8):e0330296. doi: 10.1371/journal.pone.0330296 (PMC12352851; doi:10.1371/journal.pone.0330296)
Supplement: S5 Table — PHCs, primary healthcare centers. (PDF) [file pone.0330296.s005.pdf]

S5 Table. Parallel trend test for VBP drugs.

Time	Total		Type of medical institution						Therapeutic category									
			Tertiary hospital		Secondary hospital		PHCs		C		N		L		J		others	
	Coef.	*P*-value	*Coef.*	*P*-value	*Coef.*	*P*-value	*Coef.*	*P*-value	*Coef.*	*P*-value	*Coef.*	*P*-value	*Coef.*	*P*-value	*Coef.*	*P*-value	*Coef.*	*P*-value
eventz2	-0.09	0.790	-0.27	0.423	0.10	0.773	0.29	0.401	0.03	0.941	-0.20	0.393	-0.24	0.367	-0.29	0.514	-0.19	0.657
eventz3	0.19	0.560	0.05	0.877	0.30	0.394	0.53	0.128	0.30	0.433	0.08	0.738	0.15	0.572	-0.11	0.810	0.11	0.799
eventz4	0.05	0.879	-0.04	0.893	0.16	0.646	0.30	0.386	0.15	0.691	0.03	0.881	0.09	0.720	-0.13	0.773	-0.13	0.764
eventz5	0.16	0.611	0.06	0.852	0.29	0.405	0.42	0.229	0.26	0.491	0.09	0.686	0.10	0.705	-0.02	0.968	0.10	0.816
eventz6	0.19	0.548	0.11	0.734	0.30	0.379	0.41	0.240	0.25	0.501	0.10	0.651	0.22	0.410	0.08	0.859	0.17	0.689
eventz7	0.13	0.672	0.03	0.937	0.18	0.589	0.45	0.195	0.27	0.476	0.05	0.807	0.19	0.458	-0.02	0.962	-0.04	0.917
eventz8	0.24	0.454	0.16	0.624	0.27	0.424	0.49	0.152	0.36	0.331	0.12	0.583	0.26	0.321	0.13	0.758	0.12	0.778
eventz9	0.38	0.229	0.27	0.415	0.53	0.123	0.64	0.066	0.45	0.226	0.22	0.330	0.41	0.118	0.24	0.590	0.43	0.300
eventz10	0.22	0.485	0.11	0.725	0.21	0.525	0.55	0.110	0.40	0.277	-0.13	0.555	0.32	0.215	0.17	0.701	0.05	0.905
eventz11	0.42	0.182	0.33	0.309	0.44	0.198	0.71	0.040	0.58	0.122	0.18	0.416	0.31	0.238	0.45	0.303	0.37	0.360
eventz12	0.51	0.109	0.37	0.255	0.58	0.086	0.84	0.015	0.71	0.059	0.27	0.226	0.23	0.369	0.42	0.331	0.48	0.238
eventz13	0.70	0.029	0.56	0.087	0.80	0.020	1.02	0.004	0.90	0.017	0.49	0.030	0.54	0.040	0.79	0.070	0.56	0.173
eventz14	0.03	0.925	-0.04	0.891	-0.05	0.873	0.32	0.338	0.19	0.596	-0.19	0.381	-0.07	0.781	0.14	0.738	-0.05	0.893

Note: PHCs, primary healthcare centers.
