# Supplementary material for: Pharmaceutical expenditure changes under the volume-based procurement policy: Effects and influencing factors
Source: PLoS One. 2025 Aug 14;20(8):e0330296. doi: 10.1371/journal.pone.0330296 (PMC12352851; doi:10.1371/journal.pone.0330296)
Supplement: S6 Table — PHCs, primary healthcare centers. (PDF) [file pone.0330296.s006.pdf]

S6 Table. Parallel trend test for alternative drugs.

Time	Total		Type of medical institution						Therapeutic category											
			Tertiary hospital		Secondary hospital		PHCs		C		N		L		J		Others			
	Coef.	*P*-value	*Coef.*	*P*-value	*Coef.*	*P*-value	*Coef.*	*P*-value	*Coef.*	*P*-value	*Coef.*	*P*-value	*Coef.*	*P*-value	*Coef.*	*P*-value	*Coef.*	*P*-value		
eventz2	-0.22	0.244	-0.28	0.182	-0.06	0.766	-0.27	0.181	-0.23	0.194	-0.26	0.188	-0.11	0.706	-0.13	0.666	-0.22	0.422		
eventz3	-0.08	0.676	-0.08	0.708	0.04	0.864	-0.17	0.396	-0.08	0.635	0.02	0.916	0.01	0.979	-0.07	0.814	-0.10	0.728		
eventz4	-0.26	0.174	-0.19	0.353	-0.13	0.534	-0.44	0.027	-0.27	0.122	-0.12	0.528	0.004	0.990	-0.30	0.327	-0.19	0.498		
eventz5	-0.19	0.318	-0.11	0.575	-0.08	0.712	-0.37	0.061	-0.20	0.248	-0.04	0.852	0.14	0.642	-0.26	0.398	-0.11	0.688		
eventz6	-0.23	0.213	-0.16	0.424	-0.12	0.553	-0.41	0.037	-0.25	0.159	-0.04	0.823	-0.11	0.708	-0.31	0.302	-0.13	0.638		
eventz7	-0.29	0.115	-0.24	0.226	-0.21	0.293	-0.42	0.032	-0.31	0.075	-0.16	0.392	-0.08	0.786	-0.33	0.274	-0.18	0.514		
eventz8	-0.23	0.212	-0.14	0.472	-0.20	0.312	-0.38	0.050	-0.27	0.128	0.004	0.981	-0.67	0.024	-0.22	0.470	-0.12	0.657		
eventz9	-0.18	0.336	-0.17	0.385	-0.01	0.953	-0.29	0.135	-0.17	0.312	0.05	0.800	-0.82	0.006	-0.25	0.399	-0.02	0.937		
eventz10	-0.31	0.089	-0.32	0.108	-0.21	0.291	-0.38	0.054	-0.28	0.111	-0.31	0.099	-0.83	0.005	-0.28	0.345	-0.19	0.489		
eventz11	-0.12	0.503	-0.09	0.646	-0.04	0.838	-0.22	0.248	-0.16	0.366	-0.01	0.956	-0.59	0.043	0.02	0.947	0.004	0.989		
eventz12	-0.01	0.959	0.01	0.969	0.11	0.583	-0.11	0.562	-0.04	0.827	0.03	0.862	-0.41	0.158	0.18	0.545	0.11	0.690		
eventz13	0.23	0.215	0.25	0.210	0.39	0.052	0.09	0.647	0.16	0.360	0.27	0.154	0.26	0.359	0.48	0.105	0.28	0.291		
eventz14	-0.41	0.028	-0.35	0.079	-0.37	0.063	-0.51	0.009	-0.45	0.011	-0.35	0.066	-0.01	0.978	-0.31	0.294	-0.38	0.154		

Note: PHCs, primary healthcare centers.
