# Supplementary material for: Pharmaceutical expenditure changes under the volume-based procurement policy: Effects and influencing factors
Source: PLoS One. 2025 Aug 14;20(8):e0330296. doi: 10.1371/journal.pone.0330296 (PMC12352851; doi:10.1371/journal.pone.0330296)
Supplement: S7 Table — PHCs, primary healthcare centers. (PDF) [file pone.0330296.s007.pdf]

S7 Table. Parallel trend test for all observed drugs.

Time	Total		Type of medical institution						Therapeutic category									
			Tertiary hospital		Secondary hospital		PHCs		C		N		L		J		Others	
	Coef.	*P*-value	*Coef.*	*P*-value	*Coef.*	*P*-value	*Coef.*	*P*-value	*Coef.*	*P*-value	*Coef.*	*P*-value	*Coef.*	*P*-value	*Coef.*	*P*-value	*Coef.*	*P*-value
eventz2	-0.21	0.386	-0.32	0.218	-0.06	0.822	-0.07	0.765	-0.18	0.460	-0.24	0.226	-0.34	0.126	-0.29	0.401	-0.17	0.594
eventz3	0.005	0.985	-0.05	0.842	0.09	0.721	0.09	0.687	0.03	0.899	0.03	0.874	-0.01	0.957	-0.19	0.576	0.07	0.825
eventz4	-0.15	0.516	-0.16	0.540	-0.06	0.813	-0.16	0.485	-0.14	0.551	-0.06	0.752	-0.06	0.784	-0.35	0.305	-0.13	0.679
eventz5	-0.06	0.791	-0.07	0.798	0.03	0.901	-0.07	0.755	-0.06	0.812	0.01	0.963	-0.03	0.893	-0.29	0.402	0.04	0.892
eventz6	-0.07	0.770	-0.06	0.823	0.02	0.944	-0.10	0.658	-0.09	0.716	0.01	0.957	0.01	0.969	-0.29	0.397	0.08	0.794
eventz7	-0.13	0.575	-0.14	0.567	-0.09	0.715	-0.09	0.695	-0.11	0.631	-0.07	0.705	-0.01	0.973	-0.34	0.324	-0.09	0.772
eventz8	-0.05	0.836	-0.03	0.917	-0.04	0.870	-0.05	0.823	-0.05	0.847	0.05	0.816	-0.05	0.810	-0.21	0.539	0.03	0.920
eventz9	0.05	0.815	0.02	0.923	0.18	0.459	0.06	0.778	0.04	0.871	0.11	0.561	0.06	0.786	-0.20	0.563	0.28	0.374
eventz10	-0.10	0.663	-0.13	0.603	-0.08	0.745	-0.03	0.910	-0.04	0.863	-0.24	0.214	-0.03	0.891	-0.24	0.478	-0.05	0.878
eventz11	0.09	0.683	0.09	0.720	0.12	0.639	0.13	0.576	0.10	0.658	0.06	0.737	-0.02	0.920	0.06	0.868	0.23	0.460
eventz12	0.19	0.414	0.15	0.547	0.26	0.289	0.25	0.282	0.22	0.343	0.13	0.501	-0.06	0.785	0.16	0.638	0.33	0.292
eventz13	0.40	0.088	0.36	0.151	0.51	0.042	0.43	0.063	0.41	0.083	0.36	0.067	0.33	0.133	0.48	0.150	0.43	0.173
eventz14	-0.26	0.266	-0.24	0.327	-0.30	0.225	-0.22	0.326	-0.25	0.279	-0.29	0.135	-0.18	0.396	-0.26	0.430	-0.20	0.517

Note: PHCs, primary healthcare centers.
