# Supplementary material for: Pharmaceutical expenditure changes under the volume-based procurement policy: Effects and influencing factors
Source: PLoS One. 2025 Aug 14;20(8):e0330296. doi: 10.1371/journal.pone.0330296 (PMC12352851; doi:10.1371/journal.pone.0330296)
Supplement: S8 Table — VBP, volume-based procurement; INN, international nonproprietary name; CI, confidence interval; PHCs, primary healthcare centers; ATC, anatomical therapeutic and chemical. (PDF) [file pone.0330296.s008.pdf]

S8 Table. Robustness test by standard DID estimation.

Category	VBP INNs			Alternative INNs			All observed drugs		
	**Coefficient**	***P*-value**	**95% *CI***	**Coefficient**	***P*-value**	**95% *CI***	**Coefficient**	***P*-value**	**95% *CI***
**Total**	-0.60	0.000	-0.67 to -0.52	0.10	0.001	0.04 to 0.16	-0.07	0.010	-0.12 to -0.02
**Medical institution type**									
Tertiary hospital	-0.64	0.000	-0.72 to -0.56	0.09	0.004	0.03 to 0.15	-0.09	0.000	-0.14 to -0.04
Secondary hospital	-0.72	0.000	-0.82 to -0.62	0.10	0.013	0.02 to 0.18	-0.14	0.000	-0.20 to -0.07
PHCs	-0.85	0.000	-1.00 to -0.70	0.09	0.135	-0.03 to 0.20	-0.18	0.000	-0.27 to -0.09
**Therapeutic category**									
ATC_C	-0.87	0.000	-1.01 to -0.73	0.29	0.000	0.17 to 0.41	-0.002	0.965	-0.10 to 0.10
ATC_N	-0.37	0.000	-0.51 to -0.24	-0.03	0.489	-0.11 to 0.05	-0.09	0.017	-0.15 to -0.02
ATC_L	0.13	0.250	-0.09 to 0.35	0.35	0.006	0.10 to 0.60	0.29	0.001	0.12 to 0.45
ATC_J	-0.80	0.000	-0.96 to -0.63	-0.22	0.016	-0.41 to -0.04	-0.39	0.000	-0.54 to -0.24
Others	-0.67	0.000	-0.81 to -0.52	0.02	0.798	-0.16 to 0.21	-0.18	0.010	-0.32 to -0.04

Note: VBP, volume-based procurement; INN, international nonproprietary name; *CI*, confidence interval; PHCs, primary healthcare centers; ATC, anatomical therapeutic and chemical.
