# Supplementary material for: Pharmaceutical expenditure changes under the volume-based procurement policy: Effects and influencing factors
Source: PLoS One. 2025 Aug 14;20(8):e0330296. doi: 10.1371/journal.pone.0330296 (PMC12352851; doi:10.1371/journal.pone.0330296)
Supplement: S9 Table — VBP, volume-based procurement; INN, international nonproprietary name; CI, confidence interval; PHCs, primary healthcare centers; ATC, anatomical therapeutic and chemical. (PDF) [file pone.0330296.s009.pdf]

S9 Table. Robustness test by excluding potentially confounding time points.

Category	VBP INNs			Alternative INNs			All observed drugs		
	**Coefficient**	***P*-value**	**95% *CI***	**Coefficient**	***P*-value**	**95% *CI***	**Coefficient**	***P*-value**	**95% *CI***
**Total**	-0.62	0.000	-0.66 to -0.57	0.08	0.000	0.04 to 0.11	-0.09	0.000	-0.12 to -0.06
**Medical institution type**									
Tertiary hospital	-0.71	0.000	-0.75 to -0.66	0.03	0.074	-0.003 to 0.07	-0.16	0.000	-0.19 to -0.13
Secondary hospital	-0.64	0.000	-0.70 to -0.58	0.08	0.001	0.03 to 0.13	-0.13	0.000	-0.17 to -0.09
PHCs	-0.45	0.000	-0.53 to -0.36	0.16	0.000	0.10 to 0.23	-0.02	0.497	-0.07 to 0.04
**Therapeutic category**									
ATC_C	-0.80	0.000	-0.88 to -0.73	0.24	0.000	0.17 to 0.31	-0.03	0.293	-0.09 to 0.03
ATC_N	-0.31	0.000	-0.39 to -0.22	0.08	0.001	0.03 to 0.13	0.01	0.622	-0.03 to 0.05
ATC_L	-0.03	0.622	-0.16 to 0.10	0.17	0.016	0.03 to 0.30	0.07	0.177	-0.03 to 0.16
ATC_J	-1.11	0.000	-1.21 to -1.02	-0.31	0.000	-0.41 to -0.21	-0.54	0.000	-0.62 to -0.46
Others	-0.72	0.000	-0.82 to -0.63	-0.02	0.657	-0.12 to 0.07	-0.22	0.000	-0.30 to -0.15

Note: VBP, volume-based procurement; INN, international nonproprietary name; *CI*, confidence interval; PHCs, primary healthcare centers; ATC, anatomical therapeutic and chemical.
