# Supplementary material for: Pharmaceutical expenditure changes under the volume-based procurement policy: Effects and influencing factors
Source: PLoS One. 2025 Aug 14;20(8):e0330296. doi: 10.1371/journal.pone.0330296 (PMC12352851; doi:10.1371/journal.pone.0330296)

S1 Fig. Monthly trend of drug expenditure among (A) tertiary hospital, (B) secondary hospital, and (C) PHCs in pilot cities; (D) tertiary hospital, (E) secondary hospital, and (F) PHCs in expansion regions.

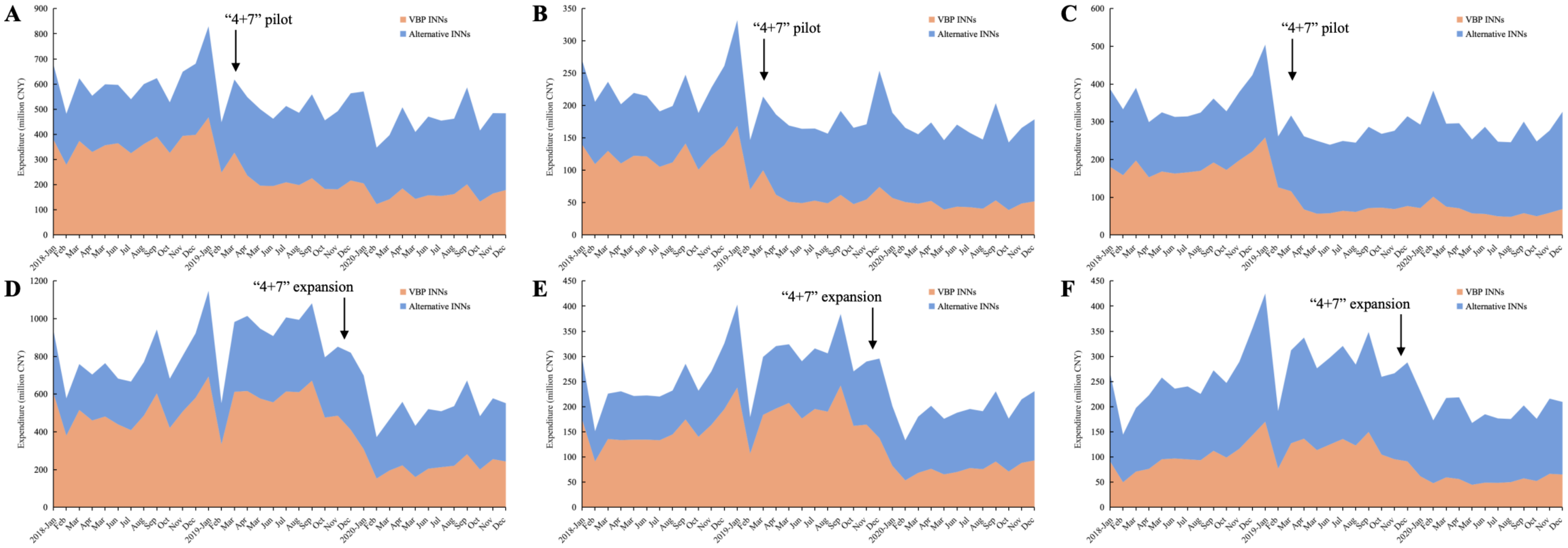

Supplement: S1 Fig — (PDF) [file pone.0330296.s010.pdf]
