# Supplementary material for: Pharmaceutical expenditure changes under the volume-based procurement policy: Effects and influencing factors
Source: PLoS One. 2025 Aug 14;20(8):e0330296. doi: 10.1371/journal.pone.0330296 (PMC12352851; doi:10.1371/journal.pone.0330296)
Supplement: Attachment — Submitted filename: Response to Reviewers.pdf [file pone.0330296.s012.pdf]

Dear Eric Nyarko,

We appreciate your suggestions and reviewers' kind comments on our submission entitled "Pharmaceutical expenditures changes under the volume-based procurement policy: effect and influencing factors" (PONE-D-25-17509). We have addressed all issues raised by the editor and reviewers and have revised the manuscript accordingly. The changes are marked with yellow highlighting in the resubmission version to facilitate the review process. Please find below the point-to-point responses.

All my best,

Ying Yang, PhD

Huazhong University of Science and Technology

Response to Journal Requirements

When submitting your revision, we need you to address these additional requirements.

1. Please ensure that your manuscript meets PLOS ONE's style requirements, including those for file naming. The PLOS ONE style templates can be found at

https://journals.plos.org/plosone/s/file?id=wjVg/PLOSOne_formatting_sample_main_body.pdf and https://journals.plos.org/plosone/s/file?id=ba62/PLOSOne_formatting_sample_title_authors_affiliations.pdf.

Response:

Many thanks to the editor for the reminder. We have checked the manuscript style when submitting revisions.

2. We note that the grant information you provided in the 'Funding Information' and 'Financial Disclosure' sections do not match.

When you resubmit, please ensure that you provide the correct grant numbers for the awards you received for your study in the 'Funding Information' section.

Response:

Thank you very much for the reminder. We confirm that the "Funding Information" section of this study is as follows (Lines 23-27 in Revised Manuscript with Track Changes):

Funding information

This study was supported by the National Natural Science Foundation of China (Grant number: 72404098), the China Postdoctoral Science Foundation (grant number: 2024M761028), the Postdoctor Project of Hubei Province (grant number: 2024HBBHCXB019), and the Postdoctoral Fellowship Program of CPSF (Grant number: GZC20240534).

3. In this instance it seems there may be acceptable restrictions in place that prevent the public sharing of your minimal data. However, in line with our goal of ensuring long-term data availability to all interested researchers, PLOS' Data Policy states that authors cannot be the sole named individuals responsible for ensuring data access (http://journals.plos.org/plosone/s/data-availability#loc-acceptable-data-sharing-methods).

Data requests to a non-author institutional point of contact, such as a data access or ethics committee, helps guarantee long term stability and availability of data. Providing interested researchers with a durable point of contact ensures data will be accessible even if an author changes email addresses, institutions, or becomes unavailable to answer requests.

Before we proceed with your manuscript, please also provide non-author contact information (phone/email/hyperlink) for a data access committee, ethics committee, or other institutional body to which data requests may be sent. If no institutional body is available to respond to requests for your minimal data, please consider if there any institutional representatives who did not collaborate in the study, and are not listed as authors on the manuscript, who would be able to hold the data and respond to external requests for data access? If so, please provide their contact information (i.e., email address). Please also provide details on how you will ensure persistent or long-term data storage and availability.

Response:

Thank you very much for the reminder. We confirm that the "Data Availability Statement" section of this study is as follows (Lines 15-20 in Revised Manuscript with Track Changes):

Data Availability Statement

The data that support the findings of this study are owned and managed by the National Health Commission (NHC) of the People's Republic of China and are not publicly available due to institutional policies. The authors do not have the authority to share the data. Data access may be granted upon reasonable request through the official website (https://www.nhc.gov.cn/) or contact number (+8610-68792114) of NHC, subject to institutional approval.

4. Thank you for stating the following in the Acknowledgments Section of your manuscript:

"This study was supported by the National Natural Science Foundation of China (Grant number: 72404098), the China Postdoctoral Science Foundation (grant number: 2024M761028), the Postdoctor Project of Hubei Province (grant number: 2024HBBHCXB019), the Postdoctoral Fellowship Program of CPSF (Grant number: GZC20240534), and the Undergraduate Innovation Program of Huazhong University of Science and Technology (Grant Number: X202410487082). The funding body played no part in the study design, collection, analysis or interpretation of data, the writing of the manuscript, or the decision to submit the manuscript for publication."

We note that you have provided additional information within the Acknowledgements Section that is not currently declared in your Funding Statement. Please note that funding information should not appear in the Acknowledgments section or other areas of your manuscript. We will only publish funding information present in the Funding Statement section of the online submission form.

Please remove any funding-related text from the manuscript and let us know how you would like to update your Funding Statement. Currently, your Funding Statement reads as follows:

"The author(s) received no specific funding for this work."

Please include your amended statements within your cover letter; we will change the online submission form on your behalf.

Response:

Many thanks to the editor for the reminder. We have included the "Funding information" in the resubmitted cover letter. As our response to comment 2 above, the "Funding information" section of this study was confirmed as follows (Lines 23-27 in Revised Manuscript with Track Changes):

> *Funding information*
>
> *This study was supported by the National Natural Science Foundation of China (Grant number: 72404098), the China Postdoctoral Science Foundation (grant number: 2024M761028), the Postdoctor Project of Hubei Province (grant number: 2024HBBHCXB019), and the Postdoctoral Fellowship Program of CPSF (Grant number: GZC20240534).*

5. Please include captions for your Supporting Information files at the end of your manuscript, and update any in-text citations to match accordingly. Please see our Supporting Information guidelines for more information: http://journals.plos.org/plosone/s/supporting-information.

Response:

Thank you very much for the reminder. We have added the “Supporting Information” section (Lines 433-454 in Revised Manuscript with Track Changes).

Response to Reviewers’ Comments

Reviewer #1: This paper summarized the changes in expenditure on policy-covered and uncovered drugs under China’s volume-based procurement and analyzed the driving factors and constraints influencing these changes. Overall, the research topic of this paper was scientific and practical, with a wide range of data and reliable analyses. The results and conclusions can provide useful references for improving the policy. The following suggestions are recommended to enhance the quality of this article:

1. Abstract lines 56-57: Relevant data should be provided.

Response:

Thank you very much for your reminder. We have made corresponding revisions in the Abstract section as follows (Line 44-46 in Revised Manuscript with Track Changes):

> *The decrease of VBP drug expenditures showed a trend of tertiary hospital (β=-0.64, p<0.001) > secondary hospital (β=-0.57, p<0.001) > primary healthcare centers (β=-0.39, p<0.001)*

2. Both the abstract and the main text mention “large hospitals” several times. What is the specific definition of this term?

Response:

Thank you very much for pointing out the issue of unclear definition. To eliminate the ambiguity, we have directly used the explicit terms “secondary and tertiary hospitals” instead of “large hospitals” in the revised manuscript. The corresponding changes are marked in Lines 45, 330, and 426 in Revised Manuscript with Track Changes.

3. Abstract: The correspondence between “policy-covered drugs and uncovered drugs” and “VBP drugs and alternative drugs” should be noted.

Response:

Thanks very much for the helpful comments. We have made some revision in the Abstract section (Line 37 in Revised Manuscript with Track Changes), trying to make the meaning clear. As follows:

> *this study included 25 policy-covered VBP drugs and 99 policy-uncovered alternative drugs as study samples.*

In addition, in the Materials and Methods section of the main text, we have added some content to clarify the relationship between "VBP drugs and alternative drugs" and "policy-covered drugs and uncovered drugs". As follows (Lines 124, 129-130 in Revised Manuscript with Track Changes):

> *(1) VBP drugs. It represents the scope of drugs covered by the VBP policy.*
>
> *(2) Alternative drugs. It represents the scope of drugs related to VBP policy but not directly covered by the policy.*

4. Abstract line 65: The conclusion states that "the VBP policy reduced drug expenditures and is beneficial in relieving patient medication burden." However, the study does not seem to have a clear corresponding indicator. The discussion section mentioned that other micro-level studies have reached similar conclusions, but this was not a direct conclusion of this study. The conclusion should be expressed more cautiously.

Response:

Thank you very much for the suggestion. To ensure the rigor of study conclusion, we have taken your suggestion to drop the phrase "relieving patient medication burden" in the revised manuscript. The corresponding changes are marked in Lines 52-54 in Revised Manuscript with Track Changes, as follows:

> *VBP policy effectively promoted the decline of total drug expenditures, primarily through manufacturer-level market displacement and the absolute price reduction.*

5. Introduction: The innovation of this study is emphasized as analyzing from the perspective of hospital drug use. Please explain in detail how this perspective differs from existing macro- and micro-level studies, and explain how analysis from this perspective can compensate for the shortcomings of existing studies, thereby more effectively supporting the objectives and significance of this study.

Response:

Many thanks to the reviewer for pointing out our oversight. We fully agree with your perspective that explicitly stating the limitations of previous studies and highlighting the distinctive aspects of the present study in the *Introduction* section is indeed crucial. We have added relevant content accordingly in the last paragraph of the *Introduction* section. As follows (Lines 96-100 in Revised Manuscript with Track Changes):

> *In summary, the large number of existing research on drug expenditures under VBP policy implementation, on the one hand, lack reliable evidence when involving the scope of drugs not covered by the policy; on the other hand, no studies have systematically investigated the elements influencing drug expenditure changes from the perspective of hospital drug use. This study tries to make up for the above two aspects.*

6. Methods: The introduction mentioned that this study was analyzed from the perspective of hospital drug use, and the discussion referred to drug expenditure as macro-level expenditure. However, the specific definition and statistical scope of drug expenditure were not clearly stated in the methods section. This should be explicitly explained in the methods section to avoid ambiguity.

Response:

We sincerely appreciate the reviewer's valuable suggestions. In response to this comment, we have added a new paragraph in the "Difference-in-difference model" section to clarify the definition of drug expenditure indicator as well as its statistical method. The corresponding modifications are highlighted in Lines 158-167 in Revised Manuscript with Track Changes:

> *The drug expenditure indicator specifically refers to medical institutions' procurement expenditures of a defined range of drugs, which is distinct from the medication costs of individual patients occurred during outpatient or inpatient visits. However, under the zero-markup policy that mandates identical procurement and retail prices for drugs in public medical institutions [25], and given procurement regulations requiring all drugs to be procured through provincial drug centralized bidding and procurement platform [26], drug procurement expenditures can essentially be considered as the aggregate sum of medication costs across all patient visits. Following the fundamental principle of expenditure equals price multiplied by quantity, the expenditure (E_{it}) of drug i during the observation period t was calculated as the product of the price (P_{it}) and quantity (Q_{it}). The formula is expressed as follows:*

$$E_{it}=\sum_{t=1}^{n}\sum_{i=1}^{n}P_{it}\times Q_{it} \tag{1}$$

7. Data sources line 125: Please provide a detailed definition of the "36 time points"?

Response:

Thanks very much for the reminder. For clarity, we have revised the relevant wording as follows (Lines 115-117 in Revised Manuscript with Track Changes):

> *This study extracted data spanning a total of 36 months from January 2018 to December 2020, with the month serving as the smallest observational time unit, encompassing 36 observation time points.*

8. Samples: The number of alternative drugs, drug generic names, drug products, and involved drug manufacturers should also be specified separately.

Response:

We thank the reviewer for pointing out this issue. We have added relevant information in the revised manuscript. As follows (Lines 139-141 in Revised Manuscript with Track Changes):

> *A total of 99 alternative INNs were included in the analysis, covering 256 drug generic names, 3523 drug products, and involving 676 drug manufacturers.*

9. Index decomposition method on drug expenditure: T0 and T1 were both stages where the policy has not been implemented. Please explain the basis for this division.

Response:

We appreciate the reviewer's reminder. The reason for setting multiple observation time points before the policy implementation was to capture the pre-policy trend and attempt to control for confounding factors beyond the VBP policy. We have added relevant content in the revised manuscript. As follows (Lines 222-227 in Revised Manuscript with Track Changes):

> *Among them, T_0 and T_1 represent the pre-intervention periods of "4+7" pilot, while T_2 and T_3 denote the post-intervention periods; T_0, T_1, and T_2 represent the pre-intervention*

periods of "4+7" expansion, with T3 marking the post-intervention period. We designed multiple observation periods prior to policy implementation to monitor pre-intervention trends and mitigate potential confounding factors beyond the VBP policy.

10. The analysis in the article involved many classifications, such as region, time, VBP drugs/alternative drugs, original drugs/generic drugs, medical institution grading, and drug ATC classification. It is recommended to describe the analysis framework, classification basis, and definitions of each category in the methods section, especially the analysis approach for subgroup analysis, to enhance the readability of the results section.

Response:

Thanks very much for the valuable comment, and we fully agree with your suggestion.

In response to this, first, we have added a detailed description of the overall analytical framework of this study, which includes considerations regarding the classification of time periods, geographical regions, and the scope of drugs. As follows (Lines 149-155 in Revised Manuscript with Track Changes):

This study adopts a comparative analysis approach overall. Firstly, in the time dimension, we divided time period before and after policy intervention to quantify changes and impacts caused by VBP policy implementation. Secondly, in the region dimension, we distinguished between "4+7" pilot cities and expansion regions, which represent two policy implementation phases with different implementation timelines, necessitating separate assessments and comparisons. Thirdly, in the drug scope dimension, we included VBP drugs and their alternative drugs, to simultaneously observe and compare the direct and indirect effects of the policy. The following methods are mainly employed.

Second, we have added the description of the subgroup analysis approach. The corresponding changes are marked in Lines 192-199 in Revised Manuscript with Track Changes:

Subsequently, subgroup analyses were conducted based on the types of medical institutions and the drug ATC categories to assess the heterogeneity of analysis results across different subgroups. We categorized public medical institutions into tertiary hospitals, secondary hospitals, and primary healthcare centers (PHCs) in accordance with China's hierarchical classification regulations for medical institutions, as differences typically exist among these institution types in terms of drug formulary, medication scale, and prescribing habits. We stratified observation drugs into distinct therapeutic areas based on the first-level ATC codes, given that drug demand and market dynamics usually vary across therapeutic areas.

11. Descriptive analysis: The location corresponding to Figure 2 should be indicated in the text.

Response:

Thank you for the reminder. We have changed accordingly. See Line 241 in Revised Manuscript with Track Changes.

12. Conclusion: The conclusion section is somewhat lengthy. It is recommended that repetitive content be deleted and that important conclusions and policy recommendations be emphasized.

Response:

Thanks very much for the helpful suggestion. We have condensed and rewritten the *Conclusion* section. As follows (Lines 421-431 in Revised Manuscript with Track Changes):

Thanks to the reviewers' suggestions, we have condensed and rewritten the conclusion section.

> *China's VBP policy substantially reduced expenditures for both policy-covered drugs and their therapeutic categories, indirectly pointing to the relief of patients' financial burden. However, spillover effects increased alternative drug spending, partially weakening the policy effect on expenditure savings. The policy impact varied regionally and institutionally, showing greater efficacy in underdeveloped areas and secondary/tertiary hospitals. Drug expenditure changes drove by multiple factors: (1) the optimization of drug use structure due to manufacturer-level market displacement (primary driver), (2) the absolute price reduction caused by VBP policy (secondary driver), and (3) utilization increases that limited policy-covered drug savings while boosting alternative drug spending. Policy improvements should adopt holistic monitoring of policy-covered and uncovered drugs to control spillovers, while promoting high-quality generic substitution to enhance market efficiency.*

Reviewer #2:

The topic is timely and policy-relevant, and the analytical framework is broadly sound.

1. While the time-varying DID model is appropriate for estimating policy effects, the lack of a clean control group and possible concurrent external shocks (e.g., the early phase of the COVID-19 pandemic) should be more explicitly discussed as threats to causal inference.

Response:

We thank the reviewer for the valuable comment. To clarify this issue, we have added relevant content about methodological limitations in the discussion section. As follows (Lines 412-420 in Revised Manuscript with Track Changes):

> *Thirdly, potential limitations regarding the efficacy of causal inference should be considered. This study employed the time-varying DID method for causal inference, selected based on the policy characteristics of staggered implementation at different time points. However, some scholars have noted limitations of the time-varying DID in estimating the average treatment effect of multi-period interventions [54,55], as its estimates can be regarded as weighted averages of multiple standard DID estimates [27]. This might lead to ambiguity in the interpretation of the time-varying DID coefficients. Although the robustness test of standard DID analyses has confirmed the reliability of the primary findings derived from time-varying DID, caution remains necessary when interpreting and comparing the estimated coefficients.*

2. The decomposition method and its interpretation are generally well explained, but additional clarification (perhaps a table or schematic) would help non-specialist readers.

Response:

Thank you very much for the suggestion. We have added Figure 1 to present the schematic diagram for the decomposition of drug expenditure change determinants. As follows:

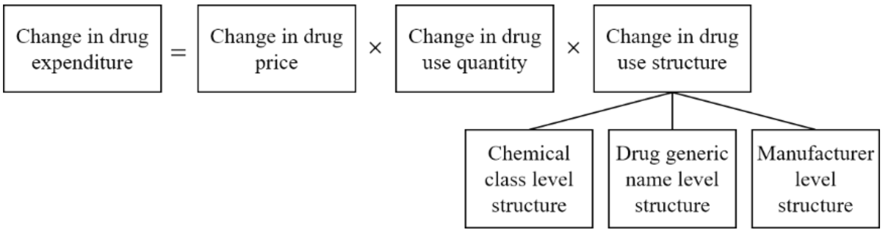

Fig 1. Schematic diagram for the decomposition of drug expenditure change determinants.

3. Consider briefly elaborating on how the alternative drugs were selected (e.g., were they matched by ATC level or based on clinical judgment?).

Response:

We thank the reviewer for the reminder. In this study, the inclusion of alternative drugs was determined following the “list of alternative drugs” provided by the National Healthcare

Security Administration (NHSA) in the *Monitoring Plan for National Centralized Volume-based Drug Procurement and Use Pilot Work*. In this process, the "list of alternative drugs" is mainly developed by clinical experts organized by the NHPA, mainly based on the clinical judgment feedback on the substitution relationship of clinical use, and also taking into account the actual procurement and use of substitution reflected by the changes in procurement data. In the revised manuscript, we have added relevant expressions in the "samples" section, marked in Lines 131-136 in Revised Manuscript with Track Changes:

> *In this study, the inclusion of alternative INNs was determined following the "list of alternative drugs" provided by the National Healthcare Security Administration (NHSA) in the Monitoring Plan for National Centralized Volume-based Drug Procurement and Use Pilot Work. The list of alternative drugs is formulated by the NHSA based on clinical substitution relationships reported by expert clinicians, while also taking into account actual procurement and use substitution patterns reflected in dynamic procurement data [24].*

4. Some figures and tables could benefit from clearer labeling (e.g., T0–T3 time periods should be defined in the figure captions themselves)

Response:

Thank you for the reviewer's reminder. It has been revised. See Lines 284-286, 638-640 in Revised Manuscript with Track Changes.

I hope the above revisions improve my manuscript, and I truly appreciate your patience and understanding. I sincerely look forward to your response.
