# Supplementary material for: Pharmaceutical expenditure changes under the volume-based procurement policy: Effects and influencing factors
Source: PLoS One. 2025 Aug 14;20(8):e0330296. doi: 10.1371/journal.pone.0330296 (PMC12352851; doi:10.1371/journal.pone.0330296)
Supplement: Attachment — Submitted filename: Review comments.docx [file pone.0330296.s011.docx]

This paper summarized the changes in expenditure on policy-covered and uncovered drugs under China’s volume-based procurement and analyzed the driving factors and constraints influencing these changes. Overall, the research topic of this paper was scientific and practical, with a wide range of data and reliable analyses. The results and conclusions can provide useful references for improving the policy. The following suggestions are recommended to enhance the quality of this article:

1. Abstract lines 56-57: Relevant data should be provided.

2. Both the abstract and the main text mention “large hospitals” several times. What is the specific definition of this term?

3. Abstract: The correspondence between “policy-covered drugs and uncovered drugs” and “VBP drugs and alternative drugs” should be noted.

4. Abstract line 65: The conclusion states that “the VBP policy reduced drug expenditures and is beneficial in relieving patient medication burden.” However, the study does not seem to have a clear corresponding indicator. The discussion section mentioned that other micro-level studies have reached similar conclusions, but this was not a direct conclusion of this study. The conclusion should be expressed more cautiously.

5. Introduction: The innovation of this study is emphasized as analyzing from the perspective of hospital drug use. Please explain in detail how this perspective differs from existing macro- and micro-level studies, and explain how analysis from this perspective can compensate for the shortcomings of existing studies, thereby more effectively supporting the objectives and significance of this study.

6. Methods: The introduction mentioned that this study was analyzed from the perspective of hospital drug use, and the discussion referred to drug expenditure as macro-level expenditure. However, the specific definition and statistical scope of drug expenditure were not clearly stated in the methods section. This should be explicitly explained in the methods section to avoid ambiguity.

7. Data sources line 125: Please provide a detailed definition of the “36 time points”?

8. Samples: The number of alternative drugs, drug generic names, drug products, and involved drug manufacturers should also be specified separately.

9. Index decomposition method on drug expenditure: T0 and T1 were both stages where the policy has not been implemented. Please explain the basis for this division.

10. The analysis in the article involved many classifications, such as region, time, VBP drugs/alternative drugs, original drugs/generic drugs, medical institution grading, and drug ATC classification. It is recommended to describe the analysis framework, classification basis, and definitions of each category in the methods section, especially the analysis approach for subgroup analysis, to enhance the readability of the results section.

11. Descriptive analysis: The location corresponding to Figure 2 should be indicated in the text.

12. Conclusion: The conclusion section is somewhat lengthy. It is recommended that repetitive content be deleted and that important conclusions and policy recommendations be emphasized.
